# Metabolic engineering of *Escherichia coli* for efficient biosynthesis of butyl acetate

**DOI:** 10.1186/s12934-022-01755-y

**Published:** 2022-02-22

**Authors:** Jason T. Ku, Arvin Y. Chen, Ethan I. Lan

**Affiliations:** 1 Department of Biological Science and Technology, National Chiao Tung University, 1001 Daxue Road, Hsinchu City, 300 Taiwan; 2 Institute of Molecular Medicine and Bioengineering, National Chiao Tung University, 1001 Daxue Road, Hsinchu City, 300 Taiwan; 3 Department of Biological Science and Technology, National Yang Ming Chiao Tung University, 1001 Daxue Road, Hsinchu City, 300 Taiwan; 4 Institute of Molecular Medicine and Bioengineering, National Yang Ming Chiao Tung University, 1001 Daxue Road, Hsinchu City, 300 Taiwan

**Keywords:** Alcohol acetyltransferase, ATF1, Butyl acetate, Butanol, Ester, Metabolic engineering

## Abstract

**Background:**

Butyl acetate is a versatile compound that is widely used in the chemical and food industry. The conventional butyl acetate synthesis via Fischer esterification of butanol and acetic acid using catalytic strong acids under high temperature is not environmentally benign. Alternative lipase-catalyzed ester formation requires a significant amount of organic solvent which also presents another environmental challenge. Therefore, a microbial cell factory capable of producing butyl acetate through fermentation of renewable resources would provide a greener approach to butyl acetate production.

**Result:**

Here, we developed a metabolically engineered strain of *Escherichia coli* that efficiently converts glucose to butyl acetate. A modified *Clostridium* CoA-dependent butanol production pathway was used to synthesize butanol which was then condensed with acetyl-CoA through an alcohol acetyltransferase. Optimization of alcohol acetyltransferase expression and redox balance with auto-inducible fermentative controlled gene expression led to an effective titer of 22.8 ± 1.8 g/L butyl acetate produced in a bench-top bioreactor.

**Conclusion:**

Building on the well-developed *Clostridium* CoA-dependent butanol biosynthetic pathway, expression of an alcohol acetyltransferase converts the butanol produced into butyl acetate. The results from this study provided a strain of *E. coli* capable of directly producing butyl acetate from renewable resources at ambient conditions.

**Supplementary Information:**

The online version contains supplementary material available at 10.1186/s12934-022-01755-y.

## Introduction

Butyl acetate is an industrially important chemical used as a solvent for various coatings and paints involved in the production of consumer products such as automobiles, wood furniture, artificial leather, printing inks, nail polish and adhesives [1–3]. In addition, it is a fruity odorant that is used as a synthetic fruit flavoring agent in food and beverages and as an odor enhancer to perfumes [4]. It is also considered as a biofuel additive for enhancing biodiesel properties [5]. These diverse applications of butyl acetate makes it a high volume production chemical with a production capacity of around 800,000 tonnes per year in Asia and 200,000 tonnes per year in the U.S. with price around $ 1.5 USD/kg [6].

Butyl acetate is currently manufactured through Fischer esterification of butanol and acetic acid under elevated temperature with sulfuric acid for catalysis [7], which is not environmentally benign for the volume at which butyl acetate is produced. To address this difficulty, mild methods for preparing butyl acetate such as using lipase for catalysis have been developed. However, lipase-catalyzed condensation requires a significant amount of organic solvent which presents another environmental challenge [8, 9]. An ideal reaction system would directly condense butanol and acetic acid in aqueous solution under mild condition. However, under mild ambient conditions, the thermodynamics for esterification is unfavorable. Previously, Atsumi group overcame this challenge by using an alcohol acetyltransferase that condenses alcohols with acyl-CoA and achieved ester production from glucose using metabolically engineered *Escherichia coli* [10]. Acyl-CoA represents an activated carboxylic acid and is synthesized through ATP-dependent ligation of coenzyme A with a carboxylic acid. This ATP-dependent activation provided the energy required for the condensation. Furthermore, using metabolically modified microorganisms to produce esters also allows for carbon feedstock flexibility as conventional carbon resources such as glucose, xylose, and glycerol have been extensively studied for various bio-productions. To date, the bio-production of isobutyl acetate [11, 12], isoamyl acetate [13, 14], ethyl acetate [15–17], geraniol acetate [18], tetradecyl acetate [10] and other esters [19–21] have been demonstrated.

**Fig. 1:**
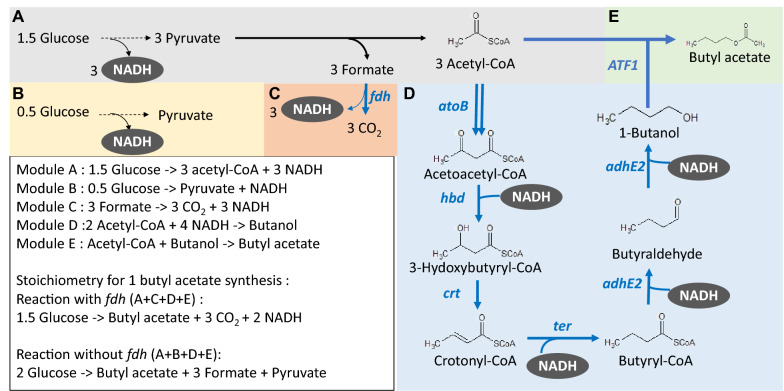
Butyl acetate production pathway from glucose. Blue arrows represent the overexpressed genes for butyl acetate production. The butyl acetate synthesis pathway is separated into **A**, **B** glycolysis, **C** formate dehydrogenase reaction, **D** butanol synthesis and **E** alcohol acetyltransferase reaction. The black box shows the stoichiometry of each pathway as well as the overall scenario of either *fdh* expressed or not. ATP equivalents are not shown in this figure

Previous studies on the production of butyl acetate have primarily been relying on feeding butanol to an alcohol acetyltransferase expressing strain [10, 22, 23]. Only a few recent studies reported direct butyl acetate synthesis from glucose in *Clostridial* strains [24, 25], which are natural butanol producers. However, as *Clostridia* are strict anaerobes with complex physiology and less tools for genetic manipulations, construction of butyl acetate producing strains using conventional hosts such as *E. coli* may offer more rapid development and easier cultivation. Therefore, here we metabolically engineered *E. coli* for direct butyl acetate production from glucose. Butyl acetate synthesis was achieved by co-expressing a modified *Clostridium* CoA-dependent butanol pathway [26] and an alcohol acetyltransferase, ATF1, from *Saccharomyces cerevisiae* s288c. Since the ATF1 protein has been reported to form aggregates in *E. coli* under high expression levels [27], we first screened for plasmids with different copy number to express *ATF1* for higher butyl acetate titer. We then investigated the stoichiometry of the whole pathway for converting glucose to butyl acetate by taking out the formate dehydrogenase (encoded by *fdh*) expression (Fig. 1), which has been previously shown to be important for butanol and butyraldehyde production [26, 28]. Finally, we replaced the IPTG-inducible promoter with self-regulated fermentation regulatory elements (FRE) [29] to construct an inducer-free butyl acetate production strain. The resulting strain was able to achieve a butyl acetate titer of 22.8 g/L using a bench-top bioreactor, representing the highest titer of butyl acetate produced by a recombinant microorganism.

## Results and discussions

### Identification of proper plasmid copy number to drive alcohol acetyltransferase expression

**Fig. 2:**
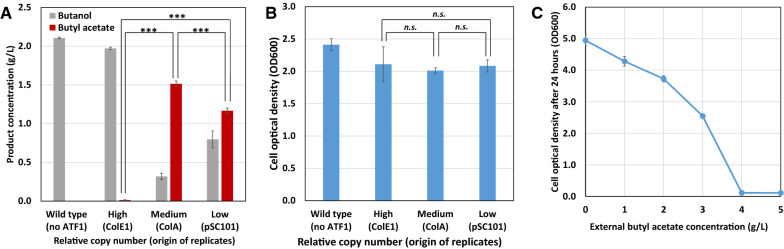
Evaluating the effect of expressing *ATF1* on different plasmid vectors for converting butanol to butyl acetate and butyl acetate toxicity to *E. coli*. **A** Product concentration in the culture broth and **B** cellular growth of *E. coli* JCL16 carrying *ATF1* gene on different copy number plasmid vectors. The cultures were incubated at 37 °C anaerobically for 24 h with butanol supplemented. The origin of replication, ColE1, ColA and pSC101 represent the relative copy number high, medium and low, respectively. **C** Growth of *E. coli* culture after 24 h with different concentration of butyl acetate added to the culture. T-test was performed to the experimental data. A *p* value larger than 0.05 is labeled as *n.s*

The most essential enzyme in developing a recombinant butyl acetate production pathway is the alcohol acetyltransferase. We previously constructed recombinant strains of *E. coli* that are efficient in producing butyraldehyde and butanol [26, 28]. Therefore, to initiate the construction of a recombinant butyl acetate producer, we focused on the expression of alcohol acetyltransferase. Alcohol acetyltransferase *ATF1* from *S. cerevisiae* has previously been selected for the production of isobutyl acetate and isoamyl acetate [12, 13] at relatively high titers. Considering the chemical similarity of butyl acetate with isobutyl acetate and isoamyl acetate, *ATF1* should be a good candidate for butyl acetate biosynthesis. First, we tested the in vivo activity of ATF1 by expressing it on a high copy plasmid with butanol feeding. Surprisingly, only trace amounts of butyl acetate were observed in the resulting culture medium, indicating minimal ATF1 activity. This result was likely due to inclusion body formation as ATF1 has previously been shown to aggregate in *E. coli* [27]. Therefore, we varied the plasmid origin of replication from high copy to medium and low copy in order to assess the effect of plasmid copy number on *ATF1* expression. We confirmed the relative copy number of *ATF1* on these vectors by qRT-PCR (Additional file 1: Fig. S1A). As shown in Fig. 2A, upon switching the expression vector from high copy to medium and low copy plasmids, recombinant *E. coli* strain was able to convert butanol to butyl acetate with no significant effect on growth (Fig. 2B). Expressing *ATF1* gene on medium copy number plasmid with ColA origin outperformed the other expression plasmids. Further analysis of protein expression using SDS-page (Additional file 1: Fig. S1B) showed that similar ATF1 expression was observed when using pSC101 and ColA origins. Therefore, subsequent experiments will express *ATF1* on a medium copy plasmid.

### Construction of recombinant butyl acetate producing strains

To achieve butyl acetate production directly from glucose, we next combined the CoA-dependent butanol production pathway, encoded by *atoB*, *hbd*, *crt*, *ter*, and *adhE2*, and the alcohol acetyltransferase, *ATF1*, into a single *E. coli* strain. Previously, we showed that the removal of competing fermentative pathways was essential for achieving high flux production of butanol [26]. Therefore, we used the same host strain JCL299 in this study for butyl acetate production. Here, we used the same three plasmid system, pEL11/pIM8/pCS138 (Table 1), previously shown to efficiently produce butanol. As the results shown in the above section, *ATF1* gene expressed on a medium copy number plasmid yielded the highest butyl acetate production. Therefore, we modified pIM8 by expressing *ATF1* together with *ter* in an operon. Furthermore, the chloramphenicol resistance gene (Cm^R^) in pCS138 was replaced by a spectinomycin resistance gene (Spec^R^) to avoid ethyl acetate production from ethanol added to medium as chloramphenicol is typically dissolved in ethanol. The resulting strain YA2 produced 0.39 ± 0.01 g/L of butyl acetate with no detectable ethyl acetate (Table 2) in a 24 h batch fermentation in test tube. Its control strain YA1 which lacks the expression of *ATF1* showed no butyl acetate production. Comparing the biochemical production profile of strains YA2 and YA1, significant differences in by-product pyruvate and formate secretion, glucose consumption, and cell growth were observed. Butyl acetate producing strain YA2 resulted in significantly slower growth with OD600 of 0.87 and is accompanied by lower glucose consumption of 8.0 ± 0.4 g/L compared to the butanol producing strain YA1. This level of growth retardation is unlikely to be due to butyl acetate toxicity as only minor inhibitory effect of butyl acetate at 0.5 g/L concentration was observed (Fig. 2C).

**Table 1:** Strains and plasmids used in this study

	Relevant genotype	References
Strain		
BW25113	*rrnB*_T14_ Δ*lacZ*_WJ16_ *hsd*R514 Δ*araBAD*_AH33_ Δ*rhaBAD*_LD78_	
XL1-blue	*recA1 endA1 gyrA96 thi*-*1 hsdR17 supE44 relA1 lac* [F’ *proAB lacI*^*q*^*ZΔM15 Tn10* (Tet^R^)]	Agilent Technologies
JCL16	BW25113/F’ [*traD36 proAB*^+^ *lacI*qZΔM15 (Tet^R^)]	[39]
JCL299	JCL16 *ΔadhE ΔldhA ΔfrdBC Δpta*	[39]
YA1	JCL299 transformed with pEL11, pIM8, pBA1	This work
YA2	JCL299 transformed with pEL11, pBA5, pBA1	This work
YA3	JCL299 transformed with pEL11, pBA5	This work
YA5	JCL299 transformed with pRW13, pYA2	This work
Plasmid		
pMW1	P_LlacO1_::*ATF1*; ColA ori; Kan^R^	This work
pMW4	P_LlacO1_::*ATF1*; ColE1 ori; Amp^R^	This work
pBA1	P_LlacO1_::*fdh*; pSC101 ori; Spec^R^	This work
pBA3	P_LlacO1_::*ATF1*; pSC101 ori; Spec^R^	This work
pBA5	P_LlacO1_::*ter*, *ATF1*; ColA ori; Kan^R^	This work
pYA2	P_adhE_::*ter, ATF1*; ColA ori; Kan^R^	This work
pRW13	P_ack_::*atoB, adhE2, crt, hbd*; ColE1 ori; Amp^R^	[29]
pRW18	P_adhE_::*fdh*; pSC101 ori; Cm^R^	[29]
pRW22	P_adhE_::*ter*; ColA ori; Kan^R^	[29]
pEL11	P_LlacO1_::*atoB, adhE2, crt, hbd*; ColE1 ori; Amp^R^	[26]
pIM8	P_LlacO1_::*ter*; ColA ori; Kan^R^	[26]
pCS138	P_LlacO1_::*fdh*; pSC101 ori; Cm^R^	[26]

**Table 2:** Butyl acetate production using different *E. coli* strains

Strain	Genes expressed				Acetate esters (g/L)		By-products (g/L)					Glucose consumption (g/L)	Cell optical density (OD600)
	Butanol biosynthesis	*ATF1*	*fdh*	Promoter used	Butyl acetate	Ethyl acetate	Ethanol	Butanol	Acetate	Pyruvate	Formate		
YA1	O		O	LlacO1	0 ± 0	n.d.	0.05 ± 0.01	1.8 ± 0.2	0.92 ± 0.08	1.6 ± 0.3	0 ± 0	9.7 ± 0.8	2.0 ± 0.2
YA2	O	O	O	LlacO1	0.39 ± 0.1	n.d.	0.05 ± 0.01	0.42 ± 0.05	1.02 ± 0.03	0.50 ± 0.08	0.38 ± 0.02	8.0 ± 0.4	0.87 ± 0.05
YA3	O	O		LlacO1	1.4 ± 0.1	n.d.	0.12 ± 0.01	0.33 ± 0.02	1.28 ± 0.04	1.4 ± 0.2	0.59 ± 0.19	12 ± 0.4	1.3 ± 0.1
YA5	O	O		FRE	1.5 ± 0.1	n.d.	0.18 ± 0.01	0.53 ± 0.04	1.42 ± 0.03	1.8 ± 0.2	0.63 ± 0.06	13 ± 0.1	1.5 ± 0.1

*n.d.* not detected

^*^FRE represents promoters of native *E. coli* fermentative genes. See Table 1 for detailed plasmid used

Next, we analyzed the stoichiometry of the butyl acetate production and compared it with butanol production hoping to identify potential causes for the reduced cell growth. As depicted in Fig. 1, when *fdh* is expressed, butyl acetate production strain results in NADH excess. Since the native fermentation pathways were deleted in strain YA2, the excess NADH generation during butyl acetate synthesis may potentially decrease the intracellular NAD^+^ concentration, leading to lower glycolysis rate and growth. To test this, we removed *fdh* from strain YA2, resulting in strain YA3. As the results shown in Table 2, strain YA3 has improved growth and glucose consumption compared to strain YA2 expressing *fdh*. Formate secretion was also increased in strain YA3 upon the removal of *fdh*. More interestingly, production of butyl acetate significantly improved to 1.4 ± 0.1 g/L. This result suggests that expression of *fdh* is less favorable for butyl acetate production and more favorable for butanol production. We reasoned that this behavior is due to *fdh* expression causing an increase in intracellular NADH levels [26]. Increased level of NADH drives acetyl-CoA flux towards butanol biosynthesis, potentially leaving less acetyl-CoA for butyl acetate production. This is in part supported by a higher butanol production by strain YA2 compared to YA3. While the production titer of butyl acetate is higher when *fdh* is not expressed, pyruvate secretion is significantly increased. This result is most likely due to a shortage of NADH. Butyl acetate production pathway is the only fermentation pathway available. 4 NADH are required for each butyl acetate synthesized and 3 NADH are produced by glycolysis to generate the 3 acetyl-CoA molecules for producing butyl acetate. The 1 NADH short is made up by oxidation of 0.5 glucose to pyruvate. Since pyruvate can be secreted from the cell while acetyl-CoA cannot, pyruvate is secreted to avoid acetyl-CoA accumulation.

### Analysis of intracellular redox state and ATP concentrations

**Fig. 3:**
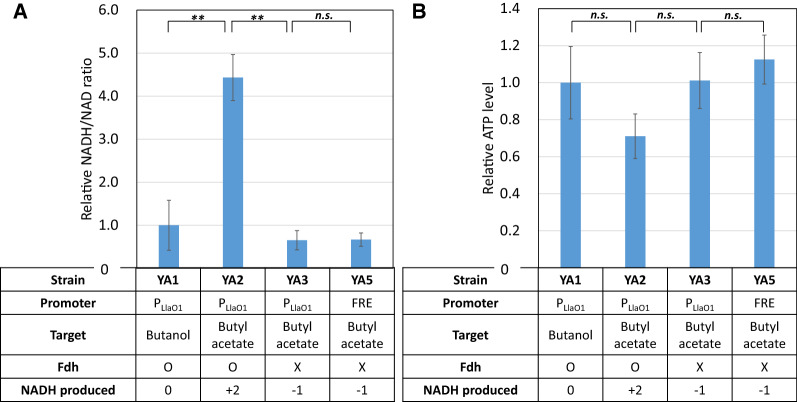
Intracellular redox level and ATP concentration. **A** Relative NADH/NAD^+^ ratio and **B** ATP level normalized by the value in butanol producing strain YA1. Positive and negative value for NADH produced represents net NADH produced for a molecule butanol or butyl acetate production. T-test was performed to the experimental data. A *p* value larger than 0.05 is labeled as *n.s.*

As described above, NADH is in excess when *fdh* is expressed, which causes a decrease in the intracellular NAD^+^ concentration, leading to lower glycolysis rate and growth. We measured the NADH to NAD^+^ ratio in each butyl acetate producing strain to investigate the relationship between butyl acetate titer and intracellular redox state. As the result shown in Fig. 3A, NADH/NAD ratio of *fdh*-expressing strain YA2 is significantly higher than that of strain YA3 which does not express *fdh*. Previous study showed that high NAD^+^ availability benefits high glycolytic rate [30]. The high NADH/NAD^+^ ratio in strain YA2 limited NAD^+^ availability, resulting in lowered glycolytic rate, potentially explaining the low butyl acetate titer, cell density, and glucose consumption in strain YA2 (Table 2). Analysis of intracellular ATP concentration (Fig. 3B) also indicated a lower glycolytic activity in strain YA2. Since phosphate acetyltransferase (Pta) was knocked out in all strains, the primary source of ATP generation is through glycolysis. Strain YA2 had the lowest ATP level when compared to butanol producing strain YA1 and butyl acetate producing strain without *fdh* YA3, potentially explaining its slower growth rate. Interestingly, we noticed that although butyl acetate production without *fdh* expression is theoretically short of NADH, it achieved a similar NADH/NAD ratio to that of the butanol producing strain YA1 which has a balanced NADH production and consumption. Strain YA3 achieved this similar NADH/NAD ratio as YA1 likely through the secretion of pyruvate. Strain YA3 secreted pyruvate with a normalized titer of 1.07 g/L/OD of while strain YA1 secreted 0.8 g/L/OD. Together, these results provided intracellular explanation to why *fdh* expression is unfavorable for butyl acetate biosynthesis.

### Using native fermentative regulatory elements to construct an inducer-free butyl acetate producing strain

Next, we wanted to establish an inducer-free butyl acetate production system to avoid the necessity of IPTG inducer which is cost prohibitive in large scale production. Since our butyl acetate production is primarily performed under anaerobic conditions, the oxygen levels and its absence can serve as a switch for expression of butyl acetate production genes. Previously, genetic sequences upstream of native *E. coli* fermentative pathway genes, including their promoters and regulatory protein binding sites, were used to drive synthetic operons for butanol production genes [29]. These sequences termed fermentative regulatory elements (FRE) enabled a higher butanol production compared to using the LlacO1 promoter and allowed inducer-free production. Here we recruited these FRE and used them on butyl acetate similar to what has been done in butanol production. Genes *atoB*, *adhE2*, *crt*, and *hbd* were grouped into a synthetic operon under the control of native acetate kinase *ack* FRE. Genes *ter* and *ATF1* were grouped into another synthetic operon under the control of native alcohol/aldehyde dehydrogenase *adhE* FRE. These two plasmids were subsequently transformed into strain JCL299, resulting in strain YA5. As the production results shown in Table 2, metabolite secretion profiles were similar to that of strain YA3 using LlacO1 promoter with IPTG as inducer. Furthermore, NADH/NAD ratio and ATP levels in strain YA5 were also similar to that of strain YA3. These results indicated the successful construction of an inducer-free butyl acetate production strain.

### Scale up butyl acetate production using bioreactor with in situ product removal

**Fig. 4:**
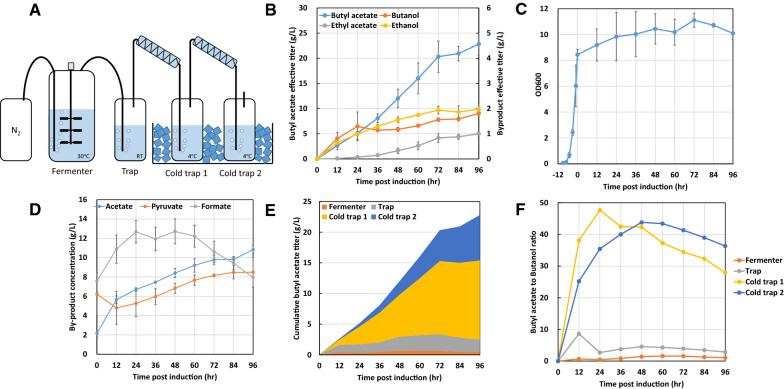
Bench-top fermentation of butyl acetate production. **A** Schematics for the fed-batch bioreactor fermentation with in situ product removal with gas striping setup. During the production phase, the bioreactor was connected to N_2_ gas cylinder to maintain the anaerobic condition. The gas outlet of the fermenter was connected to three water trap in series to capture the evaporated butyl acetate. The first water trap is placed under room temperature as indicated by RT. The second and third trap, Cold trap 1 and 2, were placed under ice bath. The Graham condenser was connected to a refrigerated chiller with circulating water at 4 °C. **B** Alcohols and ester production, **C** cellular growth, and **D** byproduct secretion profile of strain YA5 during the fermentation. **E** Butyl acetate distribution and **F** butyl acetate to butanol ratio in fermentation broth, trap (room temperature) and 2 cold traps. The time point zero indicates the time switching culture into anaerobic condition by pumping nitrogen gas

To examine the fermentation time-course behavior of the inducer-free strain YA5 for butyl acetate production, we conducted a fed-batch fermentation using a bench-top bioreactor. Since butyl acetate is toxic and inhibits *E. coli* growth at 4 g/L concentration (Fig. 2C), in situ product removal may be necessary to properly assess the butyl acetate production ability of our strain. Butyl acetate is volatile and can be removed from production vessel using gas stripping. Therefore, we used a continuous N_2_ purging fermentation coupled to water traps for product removal. Continuous bubbling of N_2_ gas carries the volatile butyl acetate out of the fermentation vessel and into sequential water traps connected by a Graham condenser. The first water trap was placed under room temperature, whereas the other two water traps were placed under ice bath. The schematics of this setup is shown in Fig. 4A. As shown in Fig. 4B, the effective titer of butyl acetate produced reached up to 22.8 ± 1.8 g/L in 96 h. The productivity for the first 72 h was nearly constant at a rate of around 0.28 g/L/hour. The cellular growth of the fermentation decreased significantly after switching to anaerobic condition for butyl acetate production (Fig. 4C). This growth behavior was similar to that reported in previous study of butanol production [26]. To our knowledge, this result represents the highest butyl acetate production titer from glucose currently reported in the literature.

Our butyl acetate production achieved a final yield of 0.12 g/g glucose, representing 37% of maximum theoretical yield. Notably, we previously showed that glycerol in TB medium has very little contribution towards product titer [26]. Therefore, it is not considered in the yield calculation. Solvent byproduct titers were about 1 order of magnitude lower than that of butyl acetate, where ethanol and butanol titer accumulated to around 2 g/L each by 96 h post anaerobic switch. Nonetheless, the notable amount of butanol secretion suggested insufficient ATF1 activity in the cell. *ATF1* expressed in *E. coli* has been previously shown with a specific activity of around 0.01 μmol/min/mg [27], while the activities of the enzymes from butanol production pathway were around 0.1 to 100 μmol/min/mg [31]. Ethyl acetate, the ATF1-catalyzed product of ethanol and acetyl-CoA, resulted in about 1 g/L. Since previous studies showed that ATF1 is more favorable on catalyzing butyl ester formation than that of ethyl ester [32, 33], the significant lower amount of ethyl acetate than butyl acetate is reasonable. Furthermore, AdhE2 enzyme is more specific towards butanol production than ethanol as it is the enzyme involved in *Clostridium* butanol fermentation [26]. No butyl butyrate was observed, indicating low activity of ATF1 for using butyryl-CoA as a substrate.

Other major byproducts were formate, acetate, and pyruvate (Fig. 4D). As the stoichiometry analysis illustrated in Fig. 1, the theoretical ratio of BA:formate:pyruvate should be 1:3:1. However, our fermentation results showed a ratio of roughly 1:0.88:0.21, showing that the expected byproducts were produced at significantly lower concentrations. This may be due to several contributing factors. First, *E. coli* contains three native formate dehydrogenases that convert formate into CO_2_ and H_2_, potentially explaining the lower formate observed in our fermentation. In particular, *fdhF* encoded an anaerobically expressed formate dehydrogenase induced by formate [34]. Second, a notable amount of carbon went to biosynthesis of butanol, ethanol, and ethyl acetate (Fig. 4B). The production of these compounds is currently inevitable due to the enzyme promiscuity of AdhE2 [35] and ATF1 proteins [10]. As ethanol and butanol are more reduced products, their biosynthesis lead to a lower stoichiometric formate production. Detailed stoichiometric balance is shown in Additional file 1: Fig. S2. Lastly, pyruvate is likely converted to acetate, which is also a major by-product in this fermentation, through acetyl-CoA. Although phosphate acetyltransferase (Pta) was knocked out, which should’ve blocked acetate formation, acetate secretion may result from non-specific activity of endogenous thioesterases in which *E. coli* has numerous homologues. Furthermore, ATF1 was previously reported to also contain thioetserase activity which can hydrolyze acetyl-CoA [36], potentially explaining the higher acetate production in this study. Formate was also produced with a high concentration up to 12 g/L, which is usually inhibitory to cell growth [37, 38]. To evaluate if formate is toxic to our culture, we measured the cell growth under different formate concentrations. The result (Additional file 1: Fig. S3) showed that formate concentration up to 15 g/L inhibited approximately 50% cell growth while 10 g/L resulted in minor inhibition. Therefore, we believe that 12 g/L of formate should not significantly affect cell growth and viability. Interestingly, while acetate and pyruvate secretion gradually increased with time, we noticed that formate started to decline after 48 h, which may due to conversion to CO_2_ and H_2_ by native formate dehydrogenases.

Initially most of the gas striped butyl acetate were found in water trap 1. As fermentation progresses, amount of butyl acetate in trap 1 remained around 3 g/L (Fig. 4E), indicating its saturation in water under room temperature. Majority of butyl acetate was found in the cold traps. By 96 h post anaerobic switch, more than 85% of the butyl acetate were in the cold traps. In particular, cold trap 1 was saturated with about 12 g/L of butyl acetate. Butyl acetate to butanol ratio in fermenter and the water traps gradually decreases (Fig. 4F). This result was due to gradual accumulation of butanol produced by our strain. Nonetheless, the butyl acetate to butanol ratio observed in fed-batch fermentation outperformed test tube production (Table 2), indicating in situ product removal helps to drive butyl acetate formation as butyl acetate is more volatile than butanol. Ethyl acetate showed a similar distribution in the in situ removal system to that of butyl acetate. Although ethanol (Additional file 1: Fig. S4A) accumulated a higher concentration in fermenter than butanol (Additional file 1: Fig. S4B), the ethyl acetate titer was significantly lower than butyl acetate (Additional file 1: Fig. S4C), indicating ATF1 is more favorable on using butanol as substrate instead of ethanol. Together, the results presented here showed an efficient conversion of glucose to butyl acetate.

## Conclusion

While most of the microbial butyl acetate synthesis available in the literature required externally added butanol or butyric acid, and have resulted in lower titers, in this study, we presented an inducer-free strain of *E. coli* for butyl acetate production directly from glucose. A proper vector with medium copy number was selected for alcohol acetyltransferase *ATF1* expression to achieve highest butyl acetate titer. In addition, we investigated the stoichiometry of NADH involved in butyl acetate synthesis by *fdh* expression. The final butyl acetate producing strain was constructed by expressing *ATF1* together with CoA-dependent butanol synthesis pathway without *fdh*. After substituting the IPTG inducible promoter with self-regulated fermentative regulatory elements to drive the gene expression, the highest butyl acetate effective titer achieved 22.8 ± 1.8 g/L in a bench top bioreactor. To our knowledge, this result represents the highest butyl acetate production titer from glucose currently reported in the literature.

## Materials and methods

### Strains and plasmids construction

Strains and plasmids used in this study are listed in Table 1. *E. coli* strain XL-1 blue was used for routine cloning. The production host strain JCL299 [39] was a strain previously developed using BW25113. All plasmids in this study were constructed by the Gibson assembly method [40] using purified PCR fragments. Plasmids pMW1 and pMW4 were constructed by replacing all the coding genes with an wild-type *ATF1* gene from *Saccharomyces cerevisiae* s288c in pIM8 and pEL11 [26]. Plasmids pBA1 were constructed by replacing Cm^R^ with Spec^R^ in pCS138. Plasmid pBA3 was constructed by replacing the *fdh* gene with an *ATF1* gene from *S. cerevisiae* s288c in pBA1. Plasmid pBA5 and pYA2 were constructed by inserting an *ATF1* gene from *S. cerevisiae* s288c genome at the downstream of *ter* in pIM8 and pRW22 with a ribosome binding site (RBS) sequence 5′-AGGAGATATACC-3′. All plasmids were verified through sequencing.

### Culture mediums and growth conditions

All *E. coli* strains were cultured at 37 °C in a rotatory shaker (250 rpm). Luria broth (LB) and LB plates (1.5% w/v, agar) were routinely used for *E. coli* cultivation unless otherwise specified. Terrific broth (TB; 12 g tryptone, 24 g yeast extract, 2.31 g KH_2_PO_4_, 12.54 g K_2_HPO_4_, 4 ml glycerol per liter of water) supplemented with 20 g/L glucose was used as complex medium for butyl acetate production. To investigate the toxicity level of butyl acetate or formate to *E. coli*, *E. coli* strain JCL16 was pre-cultured in LB overnight followed by a 1% inoculation into TB supplemented with 20 g/L of glucose and 0 to 5 g/L of butyl acetate or 0 to 15 g/L formate. When required, antibiotics were added into culture medium for selection at the following concentrations: kanamycin (Kan), 50 μg/mL; ampicillin (Amp), 100 μg/mL; spectinomycin (Spec), 50 μg/mL, tetracycline (Tet), 15 μg/mL. Cell growth was routinely determined by measuring optical density at wavelength of 600 nm (OD600) of cultures using a Biotek epoch 2 microplate spectrophotometer. Path length was adjusted to 1 cm.

### Production of butyl acetate

To culture the strains for butyl acetate synthesis, 1% (v/v) of overnight cultures in LB was used to inoculate 3 mL TB culture containing 20 g/L glucose with appropriate antibiotics in test tubes. When the cultures reached OD600 of 0.4–0.6, they were switched to anaerobic by transferring them into 10 ml BD vacutainer tubes. Head space was then purged with anaerobic gas (95% N_2_, 5% H_2_). When required, 0.3% (v/v) butanol and 1 mM IPTG were added into the cultures at the same time switching to BD vacutainer tubes for butyl acetate synthesis and the induction of protein expression, respectively. Cultures were sampled at specified times for optical density measurement and product quantification.

### Product quantification

Culture samples were centrifuged at 14,000 ×*g* for 5 min. The supernatants were then collected for product analysis. Butyl acetate and other alcohol concentrations were quantified by a Shimadzu GC-2010 gas chromatography (GC) equipped with a flame ionization discharge (FID) detector. The separation of compounds was performed by SH-Rtx-wax GC column (30 m, 0.32 mm i.d., 0.50 μm-thick film). GC oven temperature was initially held at 60 °C for 2 min and risen with a gradient of 45 °C/min until 85 °C. Then the oven temperature was kept at 85 °C for 2 min followed by a gradient of 45 °C/min until 230 °C and held for 2 min. Helium was used as the carrier gas. The injector was maintained at 225 °C, and the detector was maintained at 235 °C. 1 μL of samples was injected in split injection mode (1:15 split ratio) using 1-pentanol as the internal standard. Glucose and other organic acid concentrations were measured using a Shimadzu LC-2030C-3D equipped with a photodiode array detector and a refractive index detector. The injection volume used was 20 μL. The mobile phase consisted of 5 mM H_2_SO_4_ with a linear flow rate of 0.6 mL/min. Separation of metabolites was done by Agilent HiPlex-H (700 × 7.7 mm) organic acid analysis column maintained at 65 °C. A Bio-Rad Micro-Guard Cation H guard column (30 × 4.6 mm) was connected in front of the analysis column. Glucose and organic acid concentrations were monitored by refractive index detector and photodiode array detector, respectively. Concentration of each chemical in the collected samples was determined by standard curve constructed from GC or HPLC analysis using standard solutions. Glucose consumption was determined by subtracting the glucose concentration in samples from the concentration in original medium.

### Using quantitative real-time PCR (qRT-PCR) to determine relative plasmid copy number

Overnight cultures of strain JCL16 harboring pMW1, pBA3 or pMW4 were 1% (v/v) inoculated into 250 mL baffle flask containing 20 mL TB medium supplemented with 20 g/L glucose. 1 mM IPTG was used to induce the culture when OD600 reached 0.4–0.6. At the time of induction, the cultures were switched to anaerobic condition through purging the head space with anaerobic gas (95% N_2_, 5% H_2_). After 16 h of anaerobic cultivation, 2 mL of cultures were harvested through centrifugation at 10,000 ×*g* for 5 min and stored at − 80 °C for total DNA extraction. Total DNA of each strain was extracted using a Wizard® genomic DNA purification kit (Promega, USA). Quantitative real-time PCR (qRT-PCR) was then performed by a SensiFAST™ SYBR@ NO-ROX Kit (Meridian Bioscience, USA) using a Mic qPCR cycler (Bio Molecule Systems, Australia). Relative copy number of each plasmid was determined by their ratio to genome.

### Analysis of ATF1 expression level on different copy number plasmids

To investigate the ATF1 expression level, overnight cultures of strain JCL16 harboring pMW1, pBA3 or pMW4 were 1% (v/v) inoculated into 250 mL baffle flask containing 20 mL TB medium supplemented with 20 g/L glucose. When the cultures reached OD600 of 0.4–0.6, 1 mM of IPTG was added into the cultures to induce ATF1 expression. Then, the induced cultures were switched to anaerobic by purging head space with anaerobic gas (95% N_2_, 5% H_2_). After 12 h anaerobic cultivation, the cells were harvested by centrifugation at 3000 rpm for 15 min. The cell pellets were resuspended in 1 mL of 100 mM Tris–HCl buffer (pH 7.5). The resuspensions were then lysed through three repeat cycles of 45 s homogenization and 2 min chilling on ice using a mini-beadbeater-16 (Biospec) with 0.1 mm beads. The homogenates were used as total lysate for SDS-page. Soluble protein was prepared by taking the supernatant of the centrifuged homogenates at 10,000 ×*g* for 10 min. Protein concentration was determined by Bradford protein assay with BSA standard (BioRad). To determine protein expression level, 30 μg of total lysate and soluble protein were used for SDS-PAGE analysis using 4–20% Mini-PROTEAN® TGX Stain-Free™ Protein Gels (BioRad) in a Mini-PROTEAN® Tetra Vertical Electrophoresis Cell (Bio-Rad, USA).

### Intracellular ATP and NADH quantification

Strain YA1, YA2, YA3 and YA5 were cultured under the condition described previously for butanol and butyl acetate productionw. The cultures at the end of production were harvested by centrifugation at 10,000 ×*g* for 5 min. The cell pellets were then lysed using an EZLys™ Bacterial Protein Extraction Reagent (BioVision, USA) by directly resuspending the cell pellets in 0.5 mL of the protein extraction reagent. The resulting mixtures were then assayed using ATP Colormetric/Fluorometric Assay Kit (BioVision, USA) and NAD/NADH Quantification Colormetric Kit (BioVision, USA) according to manufacturer’s protocol to determine intracellular ATP and NADH concentrations, respectively. The ATP and NADH concentrations were normalized by the cell amounts used for lysis.

### Bioreactor culture condition

Strain YA5 was used in the fermentation of bioreactor production of butyl acetate. Fermentations were performed in BIOSTAT®A benchtop bioreactor (Sartorius stedim biotech, Aubagne, France) with a working volume of 1 L. The overnight culture from LB was first 1% (v/v) inoculated into a 250 mL shake flask containing 20 mL of TB with 20 g/L glucose and appropriate antibiotics under 37 °C, 250 rpm as pre-culture. 10 mL of the overnight pre-culture was then used to inoculate bioreactor with 1 L TB containing 20 g/L glucose and appropriate antibiotics. Dissolved oxygen (DO) during the aerobic stage was maintained above 20% with respect to air saturation by continuous bubbling 1 vvm air and adjusting the agitation speed (from 200 to 750 rpm). The cells were grown at 30 °C under aerobic conditions in batch mode until the OD600 reached about 8. Then, 0.5 vvm of nitrogen instead of the air was bubbled through the bioreactor to switch the culture condition to anaerobic and induce the expression of enzyme involved in butyl acetate production. The time at which the culture condition was switched to anaerobic corresponded to the beginning of production. After the anaerobic switch, TB medium containing 400 g/L of glucose was used as feed to maintain the glucose concentration above 10 g/L. The agitation rate was maintain at 450 rpm. The pH was automatically controlled at 6.8 by adding 2 M NaOH solution. The exhausted gases from the bioreactor was bubbled into a 2 L water trap at room temperature followed by 2 of 2 L water traps cooled by ice bath. Fermentation samples from bioreactor and 3 traps were collected to determined cell growth, alcohol concentration, ester production and glucose concentrations.

## Supplementary Information

**Additional file 1:** Additional figures. **Figure S1.** Analysis of ATF1 copy number and protein expression level. **Figure S2.** Stoichiometry analysis of relationship between organic acids and solvent products. **Figure S3.** Formate toxicity to JCL16 strain. **Figure S4.** Solvent byproduct distribution during bench-top fermentation of butyl acetate production.

## Data Availability

The datasets during and/or analysed during the current study are available from the corresponding author on reasonable request.
