# Supplementary material for: Metabolic engineering of Escherichia coli for efficient biosynthesis of butyl acetate
Source: Microb Cell Fact. 2022 Feb 22;21:28. doi: 10.1186/s12934-022-01755-y (PMC8864926; doi:10.1186/s12934-022-01755-y)
Supplement: Supplementary file 1 — Additional file 1: Additional figures. Figure S1. Analysis of ATF1 copy number and protein expression level. Figure S2. Stoichiometry analysis of relationship between organic acids and solvent products. Figure S3. Formate toxicity to JCL16 strain. Figure S4. Solvent byproduct distribution during bench-top fermentation of butyl acetate production. [file 12934_2022_1755_MOESM1_ESM.pdf]

Supporting information

Metabolic engineering of *Escherichia coli* for efficient biosynthesis of butyl acetate

Jason T. Ku [b,d], Arvin Y. Chen [b, d], and Ethan I. Lan [a, c]*

[a] Department of Biological Science and Technology and [b] Institute of Molecular Medicine and Bioengineering, National Chiao Tung University, 1001 Daxue Road, Hsinchu City, 300, Taiwan.

[c] Department of Biological Science and Technology and [d] Institute of Molecular Medicine and Bioengineering, National Yang Ming Chiao Tung University, 1001 Daxue Road, Hsinchu City, 300, Taiwan.

*Corresponding author

E-mail:

(Ethan I. Lan) ethanilan@nctu.edu.tw

(Jason T. Ku) noeidting@gmail.com

(Arvin Y. Chen) sssh10136@gmail.com

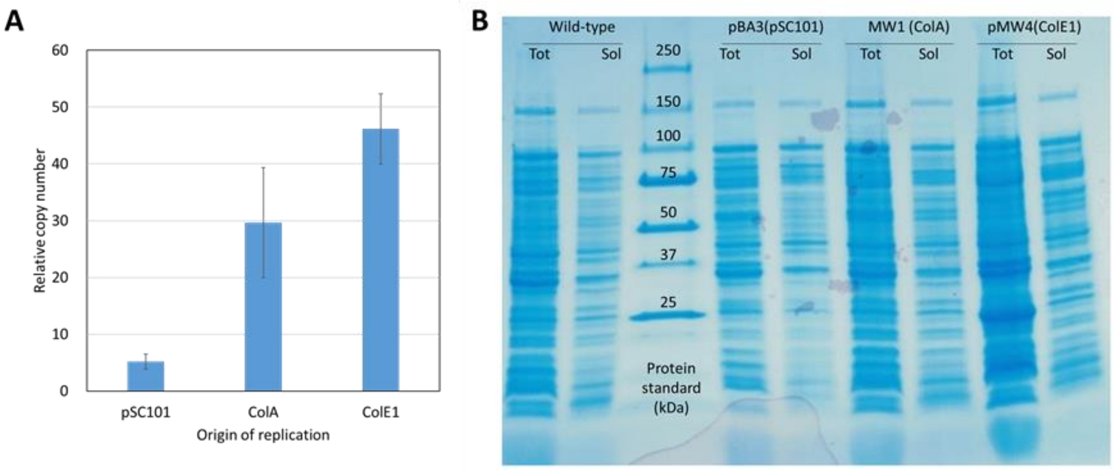

Figure S1. Analysis of ATF1 copy number and protein expression level. (A) Using qRT-PCR to determine the ATF1 copy number (B) Using SDS-page to confirm the protein expression level. Protein concentration of soluble protein extract (sol) is measured as described in method. 30 µg of soluble protein extract is used for SDS-page analysis. Total proteins (Tot) were loaded with the same volume to that of soluble protein extract. The location of ATF1 is indicated by black arrows.

Module A : 0.5 Glucose -> Pyruvate + NADH
Module B : Pyruvate -> Formate + Acetyl-CoA
Module C : Acetyl-CoA +2 NADH -> Ethanol
Module D :2 Acetyl-CoA + 4 NADH -> Butanol
Module E : Acetyl-CoA + Ethanol -> Ethyl acetate
Module F : Acetyl-CoA + Butanol -> Butyl acetate

Stoichiometry for 1 ethanol synthesis (2A+B+C) :
Glucose -> Ethanol + Formate + Pyruvate

Stoichiometry for 1 butanol synthesis (4A+2B+D) :
2 Glucose -> Butanol + 2 Formate + 2 Pyruvate

Stoichiometry for 1 ethyl acetate synthesis (2A+2B+C+E) :
Glucose -> Ethyl acetate +2 Formate

Stoichiometry for 1 butyl acetate synthesis (4A+3B+D+F) :
2 Glucose -> Butyl acetate + 3 Formate + Pyruvate

Figure S2. Stoichiometry analysis of relationship between organic acids and solvent products. To achieve net NADH balance, byproduct pyruvate for each of the solvent product synthesis from glucose is analyzed.

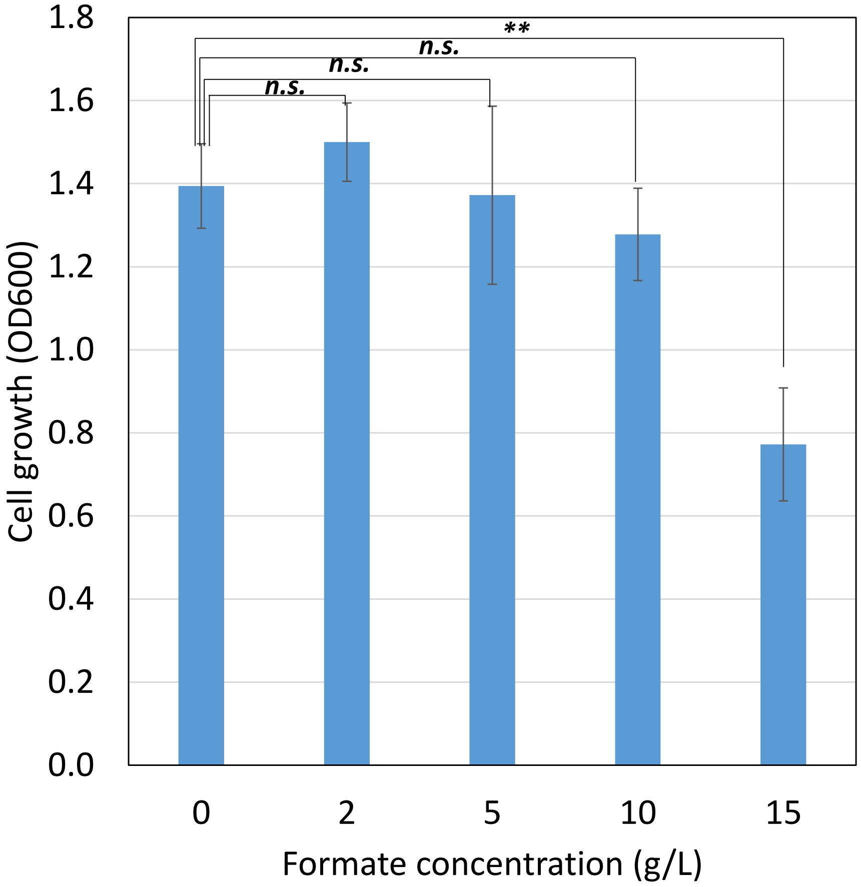

Figure S3. Formate toxicity to JCL16 strain. Overnight JCL16 strain was inoculated into TB medium supplemented with 20 g/L glucose and different concentrations of sodium formate. The cultures were then cultivated under anaerobic conditions. The cell growth was measured after 16 hr of cultivation. T-test was performed to the experimental data. A *p*-value larger than 0.05 is labeled as *n.s.*

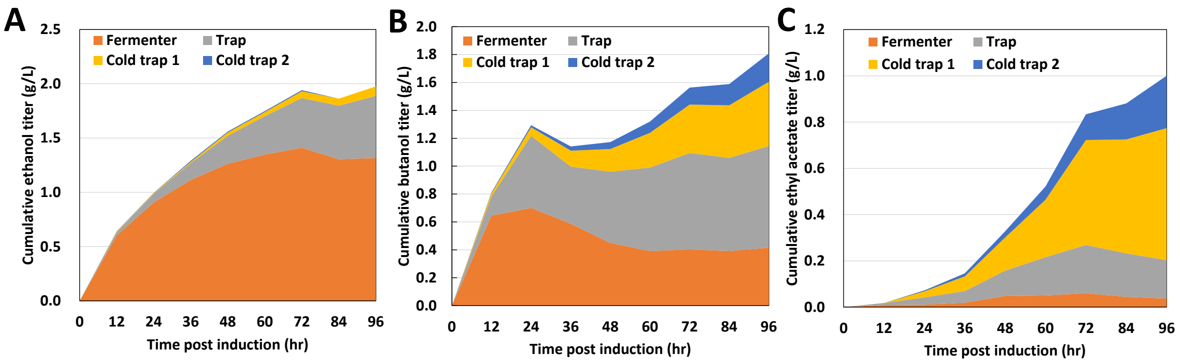

Figure S4. Solvent byproduct distribution during bench-top fermentation of butyl acetate production. (A) Ethanol, (B) butanol and (C) ethyl acetate concentrations in fermentation broth, trap (room temperature) and 2 cold traps.
